# Fermented Chinese Formula Shuan-Tong-Ling Protects Brain Microvascular Endothelial Cells against Oxidative Stress Injury

**DOI:** 10.1155/2016/5154290

**Published:** 2016-12-21

**Authors:** Lingjing Tan, Xiang Zhang, Zhigang Mei, Jinfeng Wang, Xiaoli Li, Weifeng Huang, Songbai Yang

**Affiliations:** ^1^Third-Grade Pharmacological Laboratory on Chinese Medicine Approved by State Administration of Traditional Chinese Medicine, Medical College of China Three Gorges University, Yichang, Hubei 443002, China; ^2^Key Laboratory of Cardiovascular and Cerebrovascular Diseases Translational Medicine, China Three Gorges University, Yichang, Hubei 443002, China; ^3^Yiling Hospital, Yichang, Hubei 443100, China; ^4^Yichang Hospital of Traditional Chinese Medicine, Clinical Medical College of Traditional Chinese Medicine, China Three Gorges University, Yichang, Hubei 443003, China

## Abstract

Fermented Chinese formula* Shuan-Tong-Ling* (STL), composed of fourteen medicinal herbs, was an experiential formula by Dr. Zhigang Mei for treating vascular encephalopathy, but the underlying mechanisms remained unknown. In this study, we aimed to investigate the protective effects of fermented STL on hydrogen peroxide- (H_2_O_2_-) induced injury in rat brain microvascular endothelial cells (BMECs) and the possible mechanisms. Cultured BMECs were treated with H_2_O_2_, STL, or nicotinamide (NAM, a SIRT1 inhibitor). Then, 3-(4,5-dimethylthiazol-2-yl)-2,5-diphenyl-2H-tetrazolium bromide (MTT) assay was employed to detect cell proliferation and senescence-associated beta-galactosidase (SA-*β*-gal) was used to examine cell senescence. Cell nuclei were observed by 4′,6-diamidino-2-phenylindole. Additionally, changes in reactive oxygen species (ROS), superoxide dismutase (SOD), and glutathione (GSH) levels were measured. Expression of SIRT1, p21, and PGC-1*α* was determined by western blot. Cell proliferation significantly increased with STL treatment in a dose-dependent manner. H_2_O_2_ treatment could intensify cell senescence and nuclei splitting or pyknosis. With STL treatment, the reduced ROS level was accompanied by increased SOD and GSH activity. Further assays showed upregulation of SIRT1 and PGC-1*α* and downregulation of p21 after STL treatment. The results revealed that STL could protect BMECs against oxidative stress injury at least partially through the SIRT1 pathway.

## 1. Introduction

During normal physiological conditions, reactive oxygen species (ROS) are produced at low levels and are scavenged by endogenous antioxidant systems that include superoxide dismutase (SOD), glutathione peroxidase, catalase, and small-molecule substances such as vitamins C and E [1]. But abundant accumulation of ROS may partially account for the pathogenesis of vascular diseases such as atherosclerosis, hypertension, stroke, neurodegenerative diseases, diabetes, and aging [2, 3]. It is strongly associated with endothelial dysfunction development such as endothelial injury, mitochondrial damage, inflammation, autophagy, apoptosis, and aging [4–7]. Also, ROS play an important role in mediating apoptotic death and survival in endothelial cells [8]. As a member of the brain microvascular wall, brain microvascular endothelial cells (BMECs) are the most important part of the blood-brain barrier [9] and participate in regulating vascular tone, blood fluidity and adhesiveness, and normal blood circulation [10]. Moreover, silent information regulator 1 (SIRT1) is a member of the sirtuin family of proteins, which are homologs of the Sir2 gene in* Saccharomyces cerevisiae* [11], which is highly expressed in the vasculature [12]. SIRT1 is an NAD-dependent deacetylase and modulates many biological processes, including oxidative stress, energy metabolism, cell differentiation, and genomic stability [13]. It has been reported that SIRT1 offered significant protection for cell survival in a number of disorders during oxidative stress [14, 15].

Recent studies have shown that traditional Chinese herbs or formulae, such as puerarin [16], curcumin [17], and Buyang Huanwu Decoction [18], were popularly used to attenuate oxidative stress injury and protect endothelial cells from H_2_O_2_-induced apoptosis by inhibiting oxidative stress damage or ROS-mediated mitochondrial dysfunction. In traditional Chinese medicine,* qi* is an energy that can invigorate the body and enhance blood circulation and meridian circulation [19–21]. These herbs or formulae have the functions of promoting* qi* flow and soothing liver and activating blood circulation according to the theory of traditional Chinese medicine.

*Shuan-Tong-Ling *(STL) is a new fermented Chinese formula, which is an experiential formula by Dr. Mei for treating vascular encephalopathy, which was adapted from a classical prescription, Sanpian Decoction. It is a famous traditional Chinese formula which was invented by Dr. Shiduo Chen in the Ming Dynasty for treating stroke, vascular migraine, and so forth one hundred years ago [22, 23]. STL was comprised of 14 herbs, such as Radix Puerariae* (Gegen), Salvia miltiorrhiza (Danshen),* Radix Paeoniae Alba* (Baishao),* and* Astragalus (Huangqi)*, which was fermented with* Lactobacillus*,* Bacillus aceticus*, and* Saccharomyces*. Although STL has been used to treat stroke, migraine, vascular dementia, and so on in clinic, however, the mechanism behind the beneficial effects of STL has not been explained clearly.

This study focused on determining the efficacy of STL on the H_2_O_2_-induced injury in BMECs and exploring the possible mechanisms.

## 2. Materials and Methods

### 2.1. Cell Culture

Rat brain microvascular endothelial cells (BMECs), brought from Shanghai Sxbio Biotechnology Co., Ltd., of Shanghai, were cultured in Dulbecco's modified Eagle's medium (DMEM) (Gibco, Grand Island, NY, USA) containing 15% heat-inactivated (56°C, 0.5 h) fetal bovine serum (Gibco, Grand Island, NY, USA), 100 U penicillin, and 100 *μ*g/mL streptomycin (Gibco, Grand Island, NY, USA) at 37°C in a 5% CO_2_ humidified atmosphere. Cells were seeded in culture flasks or dishes (Corning, NY, USA), grown to 75–80% confluency, and treated with H_2_O_2_ or pretreated with STL or SIRT1 inhibitor before H_2_O_2_ treatment, as stated in the following sections.

### 2.2. Preparation of STL Sample and Fermented STL

**Figure 1:**
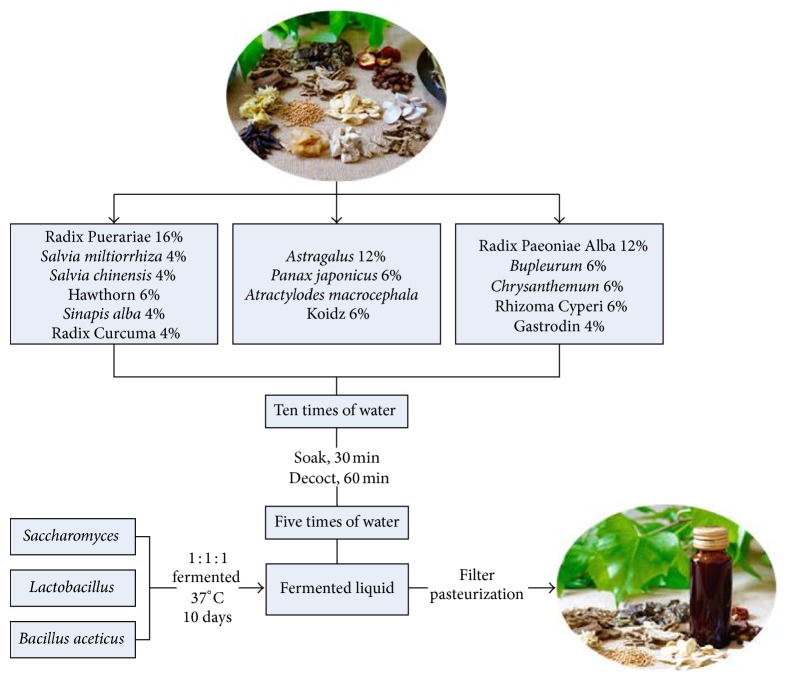
Flow chart of the manufacturing process of STL.

Fourteen herbs composing STL were obtained from Yichang Hospital of Traditional Chinese Medicine, Hubei province, China, which were classified into 3 groups based on the theory of Chinese medicine. Radix Puerariae* (Gegen)*,* Salvia miltiorrhiza (Danshen)*, Radix Curcuma* (Jianghuang)*, hawthorn* (Shanzha)*,* Salvia chinensis (Shijianchuan)*, and* Sinapis alba (Baijiezi)* were used for promoting* qi *and enhance blood circulation.* Astragalus (Huangqi)*,* Panax japonicas (Zhujieshen)*, and* Atractylodes macrocephala *Koidz* (Baizhu)* were used for invigorating* qi* and enriching blood. Radix Paeoniae Alba* (Baishao)*,* Bupleurum (Chaihu)*,* Chrysanthemum (Juhua)*, Rhizoma Cyperi* (Xiangfu)*, and* gastrodin (Tianma)* were used for modulating abnormal liquid metabolism and soothing the liver. Chopped herbs mixture at a certain weight percentage was submerged in water for 30 min and heated for 1 h at 100°C in marmite. Then, the herb decoction was fermented with* Lactobacillus*,* Bacillus aceticus*, and* Saccharomyces* for 10 days at 37°C. After sterilizing and pasteurization, final fermented liquid STL was canned and stored at 4°C (Figure 1).

### 2.3. Qualitative Analysis of Active Ingredients

Based on the theories of traditional Chinese medicine, an herbal formulation contains more than one Chinese herb. According to the literature, the effective components of STL were astragaloside IV, paeoniflorin, puerarin, curcumin, tanshinone IIA, and Chikusetsu V. These active ingredients were qualitatively controlled by high-performance liquid chromatography (HPLC) in our study. Standard chemicals including astragaloside, paeoniflorin, puerarin, curcumin, tanshinone IIA, and Chikusetsu V were purchased from Chengdu Mann Stewart Biological Technology Co. Ltd. (Chengdu, China). Briefly, HPLC profiling was performed using an Agilent 1260 series equipped with a quaternary solvent delivery system, autosampler, and a photodiode array (PDA) detector (Waters Breeze, USA). Separation was performed on an Agilent Zorbax SB-C18 column (150 mm × 4.6 mm, 5 *μ*m; temperature: 30°C, flow rate: 1 mL/min; injection volume: 10 *μ*L). The linear assessment of paeoniflorin and puerarin and curcumin gradient elution with deionized water (A) and methanol (B) was performed as follows: 0–15 min with 25–75% of B; 15–20 min with 75% of B; 20–23 min with 75–80% of B; 23–26 min with 80–25% of B. The linear assessment of Chikusetsu V gradient elution with 0.1% phosphoric acid in water (A) and methanol (B) was performed as follows: 0–10 min with 22.5% of B; 10–15 min with 22.5–40% of B; 15–20 min with 40–60% of B; 20–25 min with 60% of B; 25–30 min with 60–40% of B; 30–35 min with 40–22.5% of B. The linear assessment of tanshinone IIA gradient elution with deionized water (A) and methanol (B) was performed as follows: 0–20 min with 85% of B; 20–30 min with 85–100% of B; 30–40 min with 100–85% of B. The mobile phase of astragaloside IV was used as methanol/water (35/65), with 5-minute reequilibration of the gradient elution. The detection wavelength of astragaloside IV, paeoniflorin, puerarin, curcumin, and Chikusetsu V was 203 nm, 230 nm, 250 nm, 430 nm, 230 nm, and 270 nm, respectively.

### 2.4. Oxidative Stress Model and Treatment of the STL and Inhibitor of SIRT1

BMECs were randomly divided into four groups: (1) the control group (BMECs were cultured in normal conditions as described above); (2) the H_2_O_2_ group (BMECs were treated with H_2_O_2_ (Sinopharm Chemical Reagent Co., Ltd., Shanghai, China) at different concentrations of 0, 50, 100, 200, 400, 600, and 800 *μ*mol/L); (3) the STL group (which includes four different subgroups, in which BMECs were pretreated with final concentrations of 0, 0.0125, 0.025, 0.05, 0.1, 0.2, and 0.4 g/L, 12 h before adding H_2_O_2_ to a final concentration of 400 *μ*mol/L); (4) the SIRT1 inhibitor nicotinamide (NAM) (Beyotime, Haimen, China) group (NAM group) (BMECs were pretreated with NAM at final concentrations of 50 *μ*mol/L, 12 h before adding H_2_O_2_ to a final concentration of 400 *μ*mol/L).

### 2.5. Measurement of Cell Proliferation by MTT Assay

Cells were cultured in 96-well plates. MTT (Sigma-Aldrich, St. Louis, MO, USA) was dissolved in phosphate-buffered PBS at a concentration of 5 mg/mL. Then, the solution was filtered through a 0.2 *μ*M filter and stored at 2–8°C. At 0.5 h before the end of the equilibration, 20 *μ*L of MTT solution was added to each well; and cultures were incubated at 37°C for 4-5 h. The media were removed with a pipette, 150 *μ*L of DMSO was added to each well to dissolve the crystals, and the cell plates were covered with tinfoil, agitated on a shaker for 15 min, and finally subjected to a plate reader to read absorbance at 570 nm.

### 2.6. *β*-Galactosidase Staining

The activity of senescence-associated *β*-galactosidase (SA-*β*-gal), a marker of cellular senescence, was determined by using the Cellular Senescence Assay Kit (Beyotime, Haimen, China) according to the manufacturer's instructions. Cells were fixed and incubated with a solution containing the *β*-gal substrate, abbreviated X-gal (5-bromo-4-chloro-3-indoxyl-b-galactoside). 48 h after transfection as indicated above, cells were washed three times with PBS, fixed with 1x fixing solution, and incubated at room temperature for 10 min. After removing the fixing solution, cells were washed three times again with PBS and incubated overnight with freshly prepared 1x SA-*β*-gal detection solution at 37°C, without CO_2_, and protected from light. The percentages of blue-stained senescent cells (SA-*β*-gal-positive) were determined by counting 150 to 200 cells in five microscopic fields.

### 2.7. Immunostaining

BMECs were seeded on cover glass at a density of 1 × 10^4^ cells/well in DMEM supplemented with 10% FBS. One day after plating, cells were pretreated with STL (0.05, 0.1, and 0.2 g/L) for 12 h. The cells were then exposed to H_2_O_2_ (400 *μ*mol/L) for 24 h before incubating in fixed 4% paraformaldehyde solution. Then, cells were washed three times again with PBS and were stained with 2-(4-amidinophenyl)-6-indolecarbamidine dihydrochloride (DAPI) at 0.5 *μ*M for 30 min in room temperature. Then, cells were washed three times again with PBS. Then, cells were seeded onto glass cover slides, which were subsequently coated with glycerin. Cells were observed with 400x magnification under microscope (Leica, Germany) in five microscopic fields.

### 2.8. Measurement of Reactive ROS

Measurement of intracellular ROS was based on ROS-mediated conversion of nonfluorescent 2,7-DCFH-DA into fluorescent DCFH. Briefly, BMECs were seeded on cover glass at a density of 1 × 10^4^ cells/well in DMEM supplemented with 10% FBS. One day after plating, cells were pretreated with STL (0.05, 0.1, and 0.2 g/L) for 12 h. The cells were then exposed to H_2_O_2_ (400 *μ*mol/L) for 24 h before incubating in 2,7-DCFH-DA (Beyotime, Haimen, China) at 10 *μ*M in PBS for 30 min. Cells were washed three times with PBS and then the DCFH fluorescence from each well was excited at 488 nm and the emission was measured at 525 nm by fluorescence microscopy (Leica TCS SP5, Wetzlar, Germany). The cells were collected and observed with 400x magnification in five microscopic fields. The fluorescence of DCFH was analyzed using Image Pro plus 6.0 software (Media Cybernetics, USA).

### 2.9. Measurement of Reactive SOD and GSH

Briefly, after incubating as described above, cells were lysed and centrifuged at 12,000 ×g for 10 min at 4°C, to collect the supernatant. A commercial superoxide dismutase assay kit (Research & Diagnostic Systems, Inc., USA) and a glutathione assay kit (Research & Diagnostic Systems, Inc., USA) were used in quantification of SOD and GSH activity. The SOD and GSH levels in the supernatant were measured according to the kit protocol and analyzed on a spectrophotometer. The absorbance was read at 550 nm (SOD) and 405 nm (GSH).

### 2.10. Western Blot Analysis

BMECs were seeded into 25 × 25 cm^2^ flasks (Corning, NY, USA) and were cultured in DMEM supplemented with 10% FBS. Then, different treatments were administrated to the cells as described above. The cells were collected with a lysis buffer (Beyotime, Shanghai, China). The cleared total cell lysate was denatured via boiling and resolved via 10% sodium dodecyl sulfate-polyacrylamide gel electrophoresis. After electrophoretic separation, the proteins were transferred to a polyvinylidene fluoride membrane (Millipore, Bedford, MA, USA). The membrane was blocked with 5% skim milk for 1 h at room temperature and probed overnight with anti-SIRT1 (Santa Cruz, CA, USA; 1 : 1000), anti-PGC-1*α* (Thermo Fisher Scientific, USA; 1 : 500), anti-p21 (Cell Signaling Technology, USA; 1 : 1000), and anti-*β*-actin (Boster, Wuhan, China; 1 : 2000) at 4°C. After three washes, the blots were incubated with a goat anti-rabbit or rabbit anti-mouse peroxidase-conjugated secondary antibody (Boster, Wuhan, China; 1 : 3000) for 1 h at room temperature. The blots were displayed in Immobilon Western Chemiluminescent HRP Substrate (Boster, Wuhan, China). The protein expression levels were compared to those of *β*-actin using Quantity One software (ImageJ software, Materialise NV, Leuven, Belgium).

### 2.11. Statistical Analyses

Data are presented as mean ± standard deviation (SD). Analysis of variance was used for multiple comparisons of groups. All the analysis was carried out with GraphPad Prism 6.0 (GraphPad Software, San Diego, CA, USA). Least significant difference (LSD) analysis was used to compare the means between each of the two groups. A *P* value < 0.05 was considered statistically significant.

## 3. Results

### 3.1. Qualitative Analysis of STL Components

**Figure 2:**
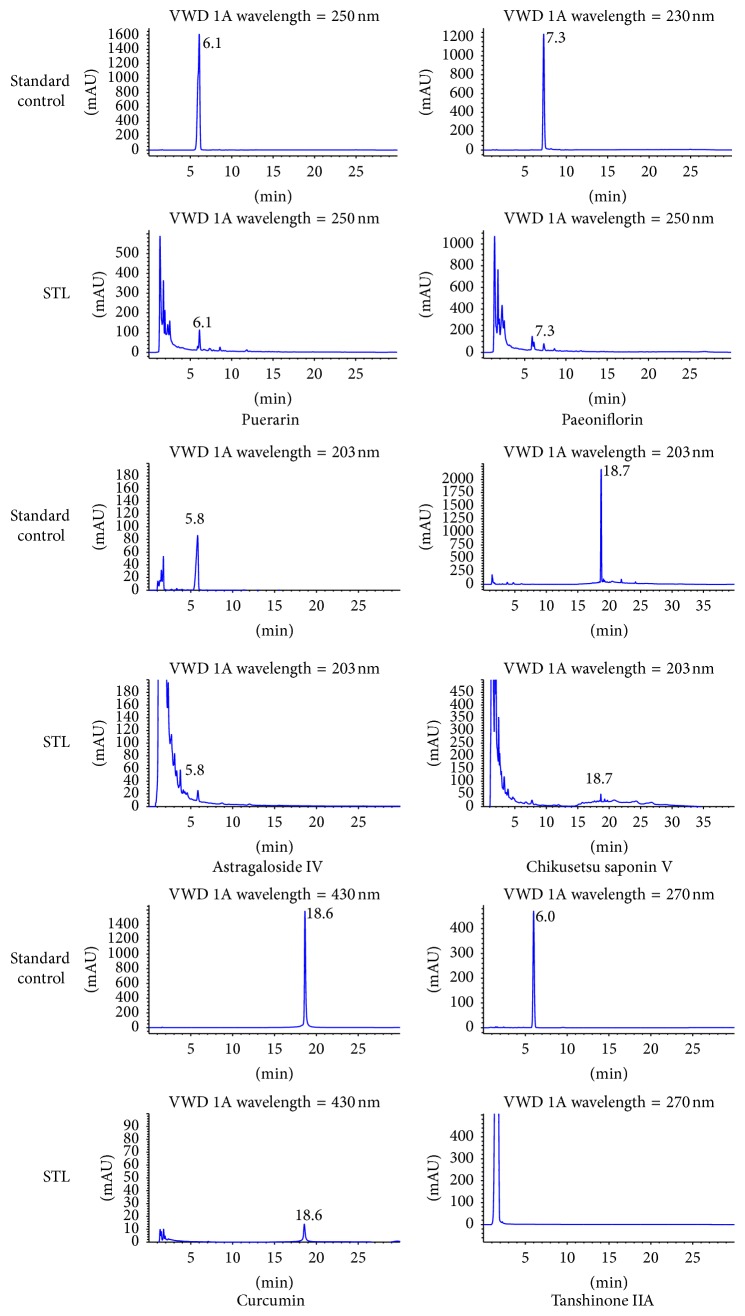
HPLC chromatograms of active ingredients in STL and standard controls. STL contained puerarin, paeoniflorin, astragaloside IV, Chikusetsu V, and curcumin, but not tanshinone IIA. The summit retention time of puerarin, paeoniflorin, astragaloside IV, Chikusetsu V, and curcumin was approximately 6.1 min, 7.3 min, 5.8 min, 18.7 min, and 18.6 min, respectively.

We used HPLC to identify the active ingredients that STL extracts contain. As shown in Figure 2, STL contained astragaloside IV, paeoniflorin, puerarin, curcumin, and Chikusetsu V, but not tanshinone IIA. The summit retention time of astragaloside IV, paeoniflorin, puerarin, curcumin, and Chikusetsu V was approximately 5.8 min, 7.3 min, 6.1 min, 18.6 min, and 18.7 min, respectively.

### 3.2. Effects of H_2_O_2_ and STL on BMECs Proliferation

**Figure 3:**
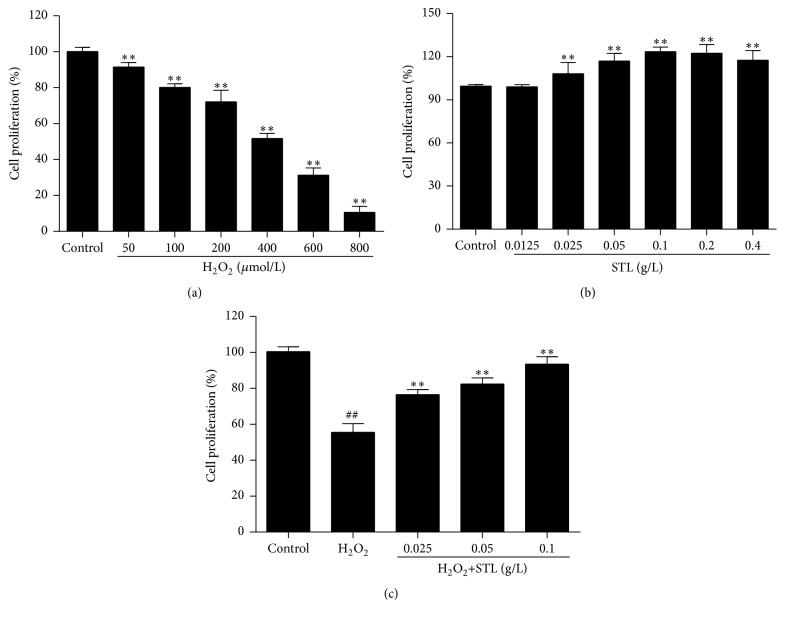
(a) Effects of H_2_O_2_ on BMECs proliferation. ^*∗∗*^ *P* < 0.01 compared to control group. (b) Effects of STL on BMECs proliferation. ^*∗∗*^ *P* < 0.01 compared to control group. (c) Effects of H_2_O_2_ and STL on BMECs proliferation. ^##^ *P* < 0.01 compared to control group. ^*∗∗*^ *P* < 0.01 compared to H_2_O_2_ group.

As shown in Figure 3(a), cell proliferation significantly decreased with H_2_O_2_ treatment in a dose-dependent manner when compared with control group (*P* < 0.01). Considering the above cell proliferation, 400 *μ*mol/L of H_2_O_2_ was chosen to induce injury in BMECs. As shown in Figure 3(b), treatment with STL at 0.0125 g/L for 12 h did not affect BMECs proliferation. However, treatment with 0.025, 0.05, and 0.1 g/L STL statistically significantly decreased cell proliferation (*P* < 0.01) when compared with control group. As shown in Figure 3(c), 400 *μ*mol/L H_2_O_2_ effectively increased the cells (*P* < 0.01) when compared with control group, while pretreatment with STL at 0.025, 0.5, and 0.1 g/L could significantly attenuate the H_2_O_2_-induced BMECs injury (*P* < 0.01) when compared with H_2_O_2_ (400 *μ*mol/L) group.

### 3.3. Effect of STL on H_2_O_2_-Induced Injury in BMECs Nuclei

**Figure 4:**
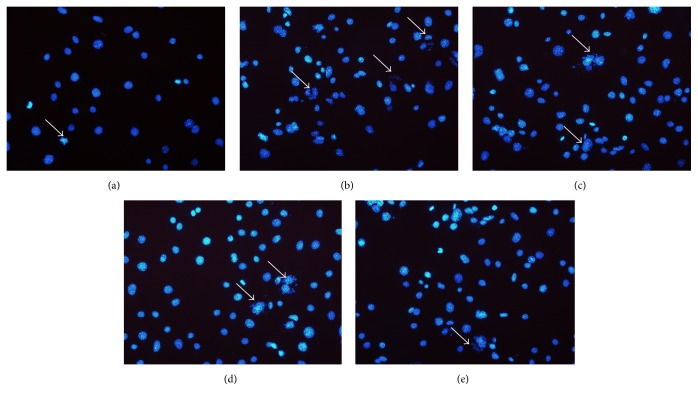
Effect of STL on H_2_O_2_-induced injury in BMECs nuclei. (a) Control group; (b) H_2_O_2_ group; (c) H_2_O_2_+STL (0.025 g/L); (d) H_2_O_2_+STL (0.05 g/L); (e) H_2_O_2_+STL (0.1 g/L).

To evaluate the effect of STL on H_2_O_2_-induced injury in BMECs nuclei, cells were stained with DAPI. Different forms of cell nuclear alterations including karyorrhexis, karyopyknosis, and karyolysis were observed after DAPI staining (blue). Considering the cell proliferation, 400 *μ*mol/L of H_2_O_2_ was chosen to induce injury in BMECs. As shown in Figures 4(a) and 4(b), exposure to H_2_O_2_ increased injury when compared with control group. As shown in Figures 4(c), 4(d), and 4(e), treatment with STL significantly reduced apoptotic cells in a dose-dependent manner when compared with H_2_O_2_ group. This suggests that STL may block BMECs nuclei injury.

### 3.4. Effect of STL on Senescence in H_2_O_2_-Induced Injury in BMECs

**Figure 5:**
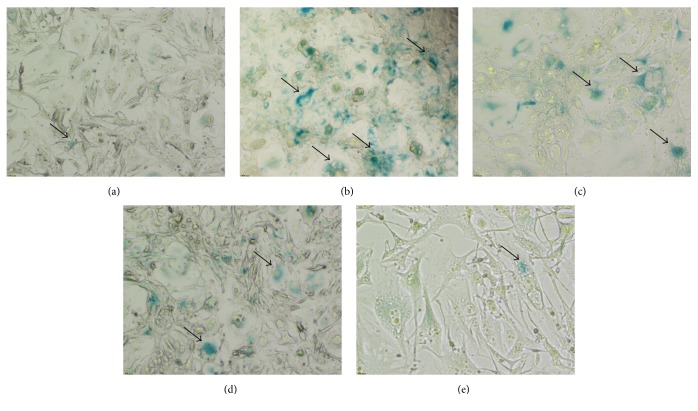
Effect of STL on senescence in H_2_O_2_-induced injury in BMECs. (a) Control group; (b) H_2_O_2_ group; (c) H_2_O_2_+STL (0.025 g/L); (d) H_2_O_2_+STL (0.05 g/L); (e) H_2_O_2_+STL (0.1 g/L).

To evaluate the effect of STL on H_2_O_2_-induced senescence, cells were stained with SA-*β*-gal. SA-*β*-gal-positive cells (blue) were considered as senescence cells. Considering the cell proliferation, 400 *μ*mol/L of H_2_O_2_ was chosen to induce injury in BMECs. As shown in Figures 5(a) and 5(b), exposure to H_2_O_2_ increased senescence when compared with control group. As shown in Figures 5(c), 5(d), and 5(e), treatment with STL significantly reduced senescence cells in a dose-dependent manner when compared with H_2_O_2_ group. This suggests that STL may block senescence pathway.

### 3.5. Effect of STL on ROS Level in H_2_O_2_-Induced Injury in BMECs

**Figure 6:**
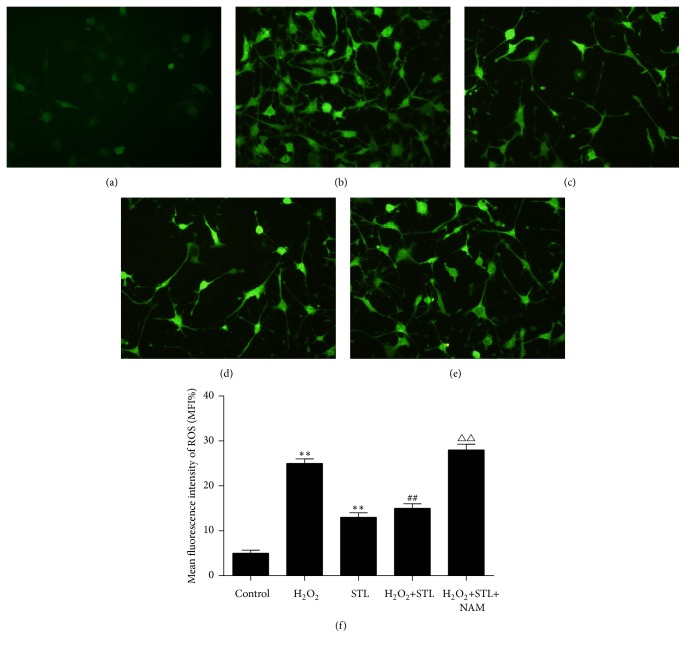
Effect of STL on ROS level in H_2_O_2_-induced injury in BMECs. (a) Control group; (b) H_2_O_2_ group; (c) H_2_O_2_+STL (0.025 g/L); (d) H_2_O_2_+STL (0.05 g/L); (e) H_2_O_2_+STL (0.1 g/L). (f) Mean fluorescence intensity of ROS. ^*∗∗*^ *P* < 0.01 compared to control group; ^##^ *P* < 0.01 compared to H_2_O_2_ group; ^△△^ *P* < 0.01 compared to H_2_O_2_+STL group.

To measure intracellular ROS levels using fluorescence microscopy with DCFH-DA, DCFH-DA is cleaved intracellularly by esterase and subsequently oxidized to DCFH by ROS. Green fluorescence produced by DCFH indicates abnormally high ROS levels. Considering the cell proliferation, 400 *μ*mol/L of H_2_O_2_ was chosen to induce injury in BMECs. As shown in Figures 6(a), 6(b), and 6(c), exposure to H_2_O_2_ decreased (*P* < 0.01) and STL group increased when compared with control group. As shown in Figures 6(d) and 6(e), treatment with STL increased ROS levels when compared with H_2_O_2_ group. This suggests that STL inhibits H_2_O_2_-induced ROS production.

### 3.6. Effect of STL on SOD and GSH Levels in H_2_O_2_-Induced Injury in BMECs

**Figure 7:**
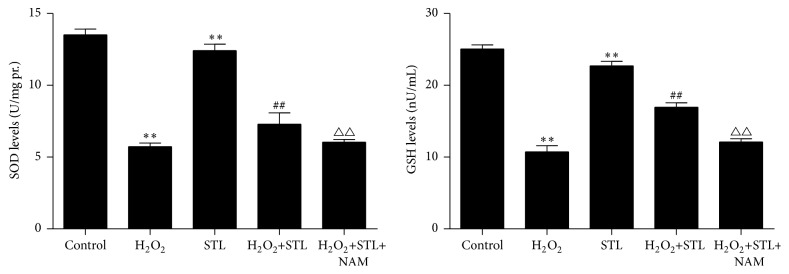
Effect of STL on SOD levels in H_2_O_2_-induced injury in BMECs. ^*∗∗*^ *P* < 0.01 compared to control group; ^##^ *P* < 0.01 compared to H_2_O_2_ group; ^△△^ *P* < 0.01 compared to H_2_O_2_+STL group. Effect of STL on GSH levels in H_2_O_2_-induced injury in BMECs. ^*∗∗*^ *P* < 0.01 compared to control group; ^##^ *P* < 0.01 compared to H_2_O_2_ group; ^△△^ *P* < 0.01 compared to H_2_O_2_+STL group.

SOD and GSH levels activity indirectly reflects the ability to eliminate ROS. To determine whether STL affects ROS-induced cell damage, we examined intracellular SOD and GSH levels. As shown in Figure 7, exposure to H_2_O_2_ decreased SOD and GSH levels (*P* < 0.01) when compared with control group and treatment with STL increased SOD and GSH levels (*P* < 0.01) when compared with H_2_O_2_ group. These findings suggest that STL prompts H_2_O_2_-induced SOD and GSH levels. Moreover, NAM treatment significantly inhibited the effects of STL (0.1 g/L) on the protein expression of SOD and GSH (*P* < 0.01) when compared with STL and H_2_O_2_ group.

### 3.7. Effect of STL on SIRT1, PGC-1*α*, and p21 Protein Expression in H_2_O_2_-Induced Injury in BMECs

**Figure 8:**
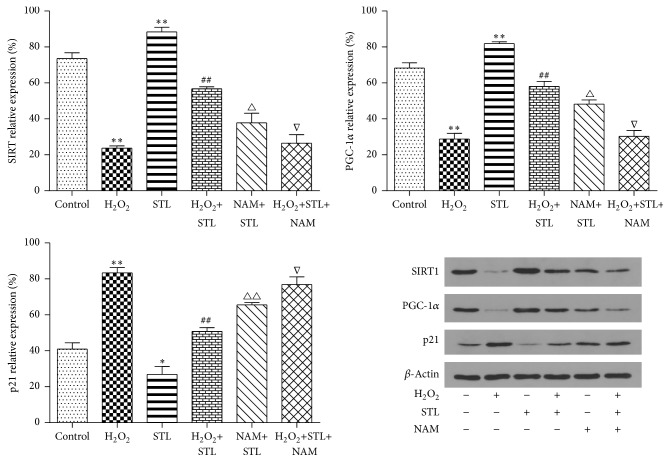
Effect of STL on SIRT1 protein expression in H_2_O_2_-induced injury in BMECs. ^*∗∗*^ *P* < 0.01 compared to control group; ^##^ *P* < 0.01 compared to H_2_O_2_ group; ^△^ *P* < 0.05 compared to STL group; ^∇^ *P* < 0.05 compared to H_2_O_2_+STL group. Effect of STL on PGC-1*α* protein expression in H_2_O_2_-induced injury in BMECs. ^*∗∗*^ *P* < 0.01 compared to control group; ^##^ *P* < 0.01 compared to H_2_O_2_ group; ^△^ *P* < 0.05 compared to STL group; ^∇^ *P* < 0.05 compared to H_2_O_2_+STL group. Effect of STL on p21 protein expression in H_2_O_2_-induced injury in BMECs. ^*∗∗*^ *P* < 0.01, ^*∗*^ *P* < 0.05 compared to control group; ^##^ *P* < 0.01 compared to H_2_O_2_ group; ^△△^ *P* < 0.05 compared to STL group; ^∇^ *P* < 0.05 compared to H_2_O_2_+STL group. Effect of STL on SIRT1, PGC-1*α*, and p21 protein expression in H_2_O_2_-induced injury in BMECs.

As shown in Figure 8, levels of SIRT1 protein expression were detected in H_2_O_2_-induced injury by western blotting. SIRT1 protein expression of H_2_O_2_-induced injury BMECs group significantly declined (*P* < 0.01) when compared to the control group. Treatment with STL increased SIRT1 protein expression (*P* < 0.01) when compared to the H_2_O_2_ group. Moreover, NAM treatment significantly suppressed the effect of STL (0.1 g/L) on the protein expression of SIRT1 (*P* < 0.05). And levels of PGC-1*α* protein expression were detected in H_2_O_2_-induced injury by western blotting. PGC-1*α* protein expression of H_2_O_2_-induced injury BMECs group was significantly downregulated (*P* < 0.01) when compared to the control group. Treatment with STL increased PGC-1*α* protein expression (*P* < 0.01) when compared to the H_2_O_2_ group. Moreover, NAM treatment significantly suppressed the effect of STL (0.1 g/L) on the protein expression of PGC-1*α* (*P* < 0.05). The levels of p21 protein expression were detected in H_2_O_2_-induced injury by western blotting. The p21 protein expression in H_2_O_2_-induced injury BMECs group was significantly upregulated (*P* < 0.01) when compared to the control group. Treatment with STL decreased p21 protein expression (*P* < 0.05) when compared to the H_2_O_2_ group. Moreover, NAM treatment significantly counteracted the effect of STL (0.1 g/L) on lowering p21 protein expression (*P* < 0.05).

## 4. Discussion

The present study suggested that STL could attenuate the pathogenic processes of H_2_O_2_-induced oxidative stress injury in rat brain microvascular endothelial cells (BMECs) by reducing H_2_O_2_-induced increase in the levels of intracellular ROS and p21 and upregulating H_2_O_2_-induced decline of GSH, SOD, and PGC-1*α* via SIRT1/PGC-*α* and SIRT1/p21 pathway.

Oxidative stress is one of the important factors leading to vascular endothelial dysfunction when endothelial cells are being exposed to excessive production of ROS and affects cell growth and apoptosis of the blood vessel walls [5]. Studies have shown that high levels of free fatty acids induced oxidative stress, which damaged human brain microvascular endothelial cells and resulted in apoptosis [24]. Oxidative stress occurs when the levels of ROS exceed the capacity of physiological antioxidant defense mechanisms [25]. Thus, overall vascular function is dependent upon the balance of oxidant and antioxidant mechanisms, which determines endothelial function. ROS consist of oxygen free radicals and associated entities that include superoxide free radicals (O_2_^−^), hydrogen peroxide (H_2_O_2_), nitric oxide (NO), and peroxynitrite (OONO^−^) [4, 26, 27]. In general, NADPH oxidase, xanthine oxidase, endothelial nitric oxide synthase (eNOS), lipoxygenases, and myeloperoxidase are the enzymes primarily involved in ROS production. Especially in the vascular system, the vascular production of ROS is mainly driven by the increased activity of the NADPH oxidase isoforms [7]. Endothelial function is usually defined as nitric oxide (NO) production and bioavailability. Because ROS can interact and inactivate NO, vascular oxidative stress can lead to a decrease in NO bioavailability. Furthermore, under the no pathological conditions, eNOS in endothelial cells produces NO by coupling with a cofactor, tetrahydrobiopterin (BH_4_). Additionally, plasma level of asymmetric dimethylarginine (ADMA), an endogenous competitive inhibitor of NO, is elevated in several diseases, including hypertension, and correlates with the degree of endothelial dysfunction. In summary, excessive ROS accumulated by increased NADPH oxidase can bring about OONO^−^ generation, ADMA inactivation, and tetrahydrobiopterin (BH_4_) oxidation, leading to reduction of NO, and then damage vascular cells [28–30].

SIRT1 is expressed throughout the body, has broad biological effects, and can significantly affect both cellular survival and longevity during acute and long-term injuries, which involve both oxidative stress and cell metabolism [31]. Research has found that SIRT1 can promote lifespan in higher organisms above yeast and metazoans [32], meanwhile promoting the protection of endothelial cells during oxidant stress exposure [14, 15]. Damaged mitochondria are thought to release more ROS and set in motion a vicious cycle of increasing DNA damage leading to increased ROS production that in turn leads to more DNA damage. Moreover, excessive accumulation of DNA damage will activate p21 and further induce cell senescence [33]. Reactive oxygen species trigger cell aging and regulate senescence-associated genes p16 and p53/p21 [34, 35]. As p21 genes are the members of the cyclin protein dependent kinase inhibitors family, cell cycle arrest is the precondition of aging, and increased p21 is enough to cause cell aging and has wide application in establishing aging model [36, 37]. SIRT1 was found to be capable of alleviating the oxidative stress-induced senescence of cartilage end plates cells in humans via downregulation of p53/p21 transcriptional activity [38] and it may be necessary for DNA repair to block apoptotic cell death during both acute injury and aging processes [39]. It is reported that SIRT1 activation can induce PGC-1*α* activity by facilitating SIRT1-mediated deacetylation and subsequently reduce peroxisome proliferator activated receptor-*α* (PPAR-*α*) activation, which results in downregulation of the NADPH oxidase subunits p22^phox^ and NOX4. SIRT1 hence plays a crucial role in reducing oxygen free radical product of blood vessels and resistance to oxidative stress [40]. PGC-1*α* is PPAR-*α* activated factor, and it can reduce oxygen free radicals induced by NADPH oxidase and increase mitochondrial DNA and raise antioxidant enzymes such as MnSOD and glutathione peroxidase enzyme (GPx1). Histone acetyltransferases compound directly acetylated PGC1-*α* multiple lysine residues and played a negative role in transcription activity [41]. In summary, SIRT1 activation will help PGC-1*α* in acetylation to combine with chromatin and increase the activity of transcription [42]. Therefore, SIRT1/PGC-1*α* signaling pathway plays a very important role in cellular defense against oxidative stress.

The origin and development of Chinese traditional medicine were based on the accumulation of lifetimes of experience and practice of the Chinese people to maintain health and treat disease. Chinese medicinal materials or prescriptions had been applied to keep balance of yin-yang and regulate qi and blood for thousands of years [43, 44]. According to the theory of traditional Chinese medicine, vascular encephalopathies, including ischemic stroke, migraine, vascular dementia, and dizziness, were usually attributed to qi deficiency, qi stagnancy, and blood stasis. As previously mentioned, the herbs in STL have the functions of invigorating and promoting qi and blood circulation and relieving depression. We used HPLC to identify the active ingredients and found that STL contained puerarin, astragaloside IV, paeoniflorin, curcumin, and Chikusetsu V. Our previous study showed that puerarin can protect against brain injury by counteracting the inflammatory response after cerebral ischemia/reperfusion via activating the cholinergic anti-inflammatory pathway [45]. Also, puerarin could provide neuroprotection against traumatic brain injury-induced oxidative stress characterized by the severe disturbance of redox balance; puerarin treatment could lead to a significant decrease in the MDA content and increase in the level of GSH in comparison with vehicle treatment after traumatic brain injury, at least in part, through the activation of PI3K-Akt pathway, which may represent a new promising therapeutic agent in the treatment of traumatic brain injury in the future [46]. Another active ingredient, astragaloside IV, had protective effects on focal cerebral ischemia in rats at different reperfusion time points. Astragaloside IV treatment increased SOD activity and decreased the level of MDA at different time points, suggesting that astragaloside IV attenuated lipid peroxidation and reduced the generation of superoxide anions in cerebral ischemia/reperfusion. The mechanism may be related to antioxidation, regulating the expressions of iNOS, NGF, and TrkA mRNA [47]. As an ingredient of STL, paeoniflorin alleviated the acute damage induced by LPS in mice brains, which was related to its antioxidant effect and improvement of energy metabolism, which could reduce the levels of MDA production and significantly increase the activities of antioxidant enzymes such as SOD and GSH-PX; besides, paeoniflorin was able to increase the expression of HO-1 and activate the nuclear transfer of Nrf2 [48]. In addition, study demonstrated that carbofuran at sublethal doses was able to cause oxidative stress damage in the brainstem of rats, with the effect being dose-dependent. But the pretreatment of animals with curcumin showed attenuation of carbofuran toxicity because the activities of SOD and GSH of rat brain were significantly increased. The ameliorative effect of curcumin may be mediated via antioxidant potential of this molecule by scavenging the free radicals [17]. Moreover, curcumin treatment at both low and high doses significantly reduced levels of ROS and MDA and enhanced the activity of SOD and GSH-PX compared with the oxyhemoglobin group, and treatment with curcumin at both low and high doses significantly reduced the levels of TNF-*α*, IL-1*β*, and IL-6 compared with the oxyhemoglobin group, suggesting that curcumin is protective against oxyhemoglobin induced neurotoxicity in an in vitro subarachnoid hemorrhage model and the protective effects of curcumin were mainly dependent on its attenuating oxidative stress, inflammation, and neuronal apoptosis [49]. Additionally, study showed that Chikusetsu V exhibited neuroprotective effects in human neuroblastoma cells by reducing cytotoxicity, scavenging oxygen free radicals, and preventing decline in mitochondrial membrane potential. It is possibly mediated by enforcing endogenous antioxidant gene expressions of SIRT1, PGC-1*α*, and Mn-SOD. As a result, antioxidant effect may be a major mechanism for Chikusetsu V-mediated neuroprotection and Chikusetsu V could be a candidate for the treatment of oxidative stress-induced neurodegenerative disease [50]. Taken together, these findings help us to enhance our understanding of how fermented STL work.

It is reported that fermentation is a slow decomposition process of organic substances induced by microorganisms. It actively ruptures the cells of herb exposing it more to the menstruum, and bacteria have enzymes that break down cell walls to further assist in the leaching process. Furthermore, fermentation is an energy yielding process in which a nutrient molecule such as glucose is broken down without oxidation [51]. Recent study has shown that fermented Chinese traditional medicine was safe and nontoxic [52], and it can be used for antitumor [53] and antifungal [54] purposes and can improve neuroprotection [55] especially against antioxidative stress [56]. It has been reported that a human umbilical vein endothelial cell (EC) culture system was used to evaluate the effects of the fermented culture broth of a traditional Chinese medicine,* Antrodia camphorata* (FCBA), against the oxidative cell damage induced by the free radical generator AAPH. FCBA treatment significantly inhibited AAPH apoptotic cell death in the ECs, as evidenced by reducing DNA fragmentation, cytochrome C release, caspase-3 activation, and dysregulation of Bcl-2 and Bax. Moreover, the AAPH-induced reductions in EC SOD activity and protein levels were prevented by FCBA [56]. These studies suggested that we can improve the antioxidant ability of traditional Chinese medicine via applying fermentation technology.

In summary, using the liquid-state fermentation method, we screened out a new dosage form of Chinese formula called* Shuan-Tong-Ling *(STL), which was adapted from a classical prescription, Sanpian Decoction, clinically employed to treat migraine and other vascular encephalopathies for hundreds of years in China. In the present study, we have shown that STL had protective effects against oxidative stress injury induced by H_2_O_2_ in BMECs, which may be via regulating SIRT1/PGC-*α* and SIRT1/p21 signaling pathway.
